# Hierarchical bayesian fusion of inspection and monitoring data for probabilistic bridge deterioration assessment

**DOI:** 10.1038/s41598-026-36808-4

**Published:** 2026-01-21

**Authors:** Benyu Wang, Ke Chen, Bingjian Wang

**Affiliations:** Department of Bridge and Tunnel Research Center, Research Institute of Highway, Ministry of Transport, Beijing, 100088 China

**Keywords:** Hierarchical bayesian inference, Bridge health monitoring, Data fusion, Bridge deterioration model, Predictive maintenance, Engineering, Mathematics and computing

## Abstract

Bridges are susceptible to long-term deterioration due to environmental exposure and cyclic loading, making the accurate evaluation of crack evolution crucial for predictive maintenance and structural safety management. Traditional deterioration models that rely solely on periodic inspection data often fail to capture the dynamic and stochastic nature of crack propagation. To address this limitation, this study proposes a hierarchical Bayesian inference framework that integrates discrete inspection data with continuous crack monitoring data to achieve a unified probabilistic characterization of bridge deterioration. First, a Bayesian Accelerated Failure Time (AFT) model based on the Weibull distribution is developed to model the failure risk of bridge deck slabs. The model robustly handles right-censored data through sampling and incorporates multiple covariates, including crack number, type, damage category, bridge span, and geometric parameters. Posterior distributions of the shape and scale parameters, together with hazard ratio analysis, quantitatively reveal the influence of each factor on structural failure risk. Second, within the dynamic state layer of the proposed hierarchical framework, a Metropolis–Hastings-based regression model is constructed to estimate incremental crack growth, which is then aggregated monthly to form a continuous degradation trend series. This method effectively captures the response of crack development to environmental fluctuations and supports predictive analysis for on-site maintenance planning. Finally, the failure risk model and the crack evolution model are coupled within a hierarchical Bayesian framework to enable joint risk estimation. The proposed model integrates multi-source information with varying temporal resolutions and uncertainty levels and employs a Bayesian posterior updating mechanism to adaptively refine parameters as new monitoring data become available. Validation using real bridge monitoring datasets demonstrates that the posterior-updated model significantly outperforms traditional inspection-based approaches in capturing crack failure behavior and long-term deterioration trends.

## Introduction

 With the rapid advancement of bridge health monitoring technologies, multi-source time-series data—such as crack width, temperature, and vibration measurements collected from field-deployed sensors—have created unprecedented opportunities for in-depth understanding of structural degradation mechanisms^1–3^However, a key challenge remains: how to construct a modeling framework that effectively captures complex dependencies among multiple variables under large-scale, incomplete, and censored observational conditions, which are commonly observed in long-term infrastructure monitoring studies^4,5^, while maintaining interpretability for engineering decision-making.

Bayesian inference provides a rigorous theoretical foundation that enables the natural integration of heterogeneous random variables (discrete and continuous) within a node–edge topology, thereby revealing causal and statistical dependencies through conditional probability distributions^6,7^.Nevertheless, conventional Bayesian models often fall short in representing complex, high-dimensional degradation systems. In contrast, hierarchical Bayesian models have been shown to effectively integrate multi-level influencing factors while explicitly accounting for parameter uncertainty and data heterogeneity^8–10^.—such as crack number, crack type, damage mode, and environmental temperature—into a unified probabilistic network. By combining prior and likelihood information, the model achieves efficient joint posterior inference, allowing both global risk quantification and local degradation tracking.

### Novelty and contributions

Despite extensive studies on probabilistic bridge deterioration modeling, most existing methods still treat inspection-based and monitoring-based data separately, limiting their ability to represent continuous degradation processes under uncertainty. This study advances the state of the art through the following key contributions:

#### Hierarchical bayesian fusion framework

A novel three-layer hierarchical Bayesian model is developed to probabilistically integrate discrete inspection records and continuous monitoring data within a unified degradation framework. This enables dynamic posterior updating as new data become available.

#### Dual-Source risk representation

The proposed method explicitly couples the inspection-derived deterioration risk (macro-level) with monitoring-based crack evolution (micro-level), thereby bridging long-term reliability analysis and short-term degradation tracking.

#### Adaptive posterior updating mechanism

By employing Metropolis–Hastings sampling within the hierarchical model, the approach adaptively refines deterioration parameters, allowing the model to continuously learn from incoming monitoring data.

#### Real-Bridge validation and predictive improvement

The framework is validated using long-term monitoring and inspection data from the Fenghua River Bridge. Results show a 23.6% improvement in predictive accuracy and enhanced sensitivity to early degradation signals compared with traditional Weibull-AFT models.

## Literature review and main contributions

Data fusion technologies were initially developed for military applications but have rapidly expanded into non-military domains due to their ability to integrate heterogeneous information sources for more accurate and robust state estimation. In the field of bridge health monitoring (BHM), multi-sensor data fusion plays a pivotal role by enabling a comprehensive assessment of structural integrity through the combination of information from various sensing modalities.

### Advances and applications of data fusion in BHM

Early fusion approaches were primarily based on statistical and information-theoretic frameworks, with the Dempster–Shafer (D-S) evidence theory being one of the most representative examples. Originally proposed by Dempster and later refined by Shafer^11^, this theory allows data fusion without requiring prior probability information. On this basis, cloud model–based fuzzy fusion was developed by Li^12,13^to replace prior information with statistical characteristics. Luo et al. (2022)^14^ integrated the cloud model with response surface methods to construct a damage identification framework capable of quantifying the similarity between structural health states under uncertainty. Expanding on the cloud model, Yao et al. (2023)^15^ incorporated it into neural network frameworks to strengthen nonlinear feature representation and achieve more accurate structural damage identification in the presence of uncertainty. These statistically weighted fusion methods have demonstrated value in BHM applications. However, conventional weighted fusion typically assumes known inter-sensor correlations (i.e., covariance matrices), which are rarely available in practice. To mitigate this limitation, the Bar-Shalom–Campo formula and its derivative, Covariance Intersection (CI) fusion^16^, were introduced to enable consistent fusion without requiring prior correlation information.

In most existing research, monitoring and inspection systems are treated as two independent subsystems within bridge evaluation frameworks, where monitoring provides real-time performance assessment and inspection provides periodic condition assessment. Yet, since both reflect different aspects of the same structural system, integrating their information streams can yield a more holistic understanding of bridge health.

Current research on integrated evaluation methods for bridge monitoring and inspection can be broadly categorized into two directions:

(1) Multi-criteria evaluation approaches—These rely on methods such as D-S evidence theory and fuzzy mathematics to assess overall bridge performance, considering aspects such as safety, functionality, and durability. For instance, Huang et al.^17,18^developed a multi-level evaluation framework for concrete-filled steel tubular arch bridges, incorporating uncertain matrices to capture interdependencies and fuzziness. Zhang et al.^19^ combined the cloud model with D-S theory to evaluate bridge cable health by converting inspection and monitoring data into membership degrees. Li^20^ fused online monitoring with manual inspection data to achieve comprehensive condition evaluation of tied-arch bridges. Despite their effectiveness, these approaches rely heavily on expert scoring or pre-defined condition grades, leading to strong subjectivity and limited scalability.

(2) Predictive modeling approaches—These utilize historical inspection and monitoring data to establish complex association or mapping models for forecasting future bridge conditions. Xia et al.^21^ constructed a neural-network-based degradation model using historical features to predict future deterioration and the effects of maintenance actions. Qiao et al.^22^ employed multiple machine learning algorithms to build nonlinear mappings between component-level and system-level condition indices. Although these data-driven methods enhance predictive capability, their performance strongly depends on the completeness and representativeness of historical datasets. Recent studies have shown that hierarchical Bayesian networks offer strong capabilities for modeling structural deterioration and capturing uncertainty^23^. Dai et al. (2022)^24^ used conditional bivariate gamma processes with Bayesian hierarchical models to describe stochastic dependencies in wheel wear degradation. Liu and Lu (2023)^25^ applied similar models to quantify uncertainty in fatigue load mixtures, demonstrating their robustness for complex systems. Goplerud (2024)^26^ found that hierarchical models can match the predictive performance of deep learning methods while remaining interpretable.

### Deterioration models

In countries such as the United States, where bridge construction and inspection practices began earlier, comprehensive bridge condition rating (CR) databases have been established, providing a solid foundation for deterioration prediction studies. Consequently, much of the existing research focuses on constructing and optimizing bridge deterioration models (DMs) that describe the evolution of CR over time^27^.

Early deterioration models primarily relied on empirical mathematical formulations, such as exponential and polynomial models^28^, to approximate degradation trends. Although these models effectively captured the general tendency of condition decline under simplified assumptions, their inherent rigidity made it difficult to represent the complex, nonlinear deterioration behavior of bridges influenced by traffic loading, environmental exposure, and material aging.

To overcome these limitations, subsequent studies introduced probabilistic and stochastic deterioration models, including the Weibull distribution model and the Cox proportional hazards (Cox-PH) model^29–31^. These models account for multiple influencing factors—such as service age, traffic intensity, and maintenance history—and allow quantitative estimation of failure probability or remaining service life.

However, the parametric assumptions in these traditional models still restrict their ability to capture state transitions, path dependencies, and environmental fluctuations that characterize real bridge deterioration. With the increasing availability of computational power and the need for uncertainty quantification^32^, researchers have begun employing Markov Chain–based models^33^ and hybrid frameworks that combine Monte Carlo Simulation (MCS) with Bayesian Networks (BN)^34,35^. These models simulate numerous potential deterioration trajectories and dynamically update bridge condition states within a Bayesian inference framework, enabling refined characterization of lifecycle deterioration and uncertainty propagation caused by environmental randomness and varying operational conditions.

### Research gap and aim

Despite the progress achieved by the aforementioned studies^27–35^, a unified probabilistic framework that jointly fuses discrete inspection records and continuous monitoring data while enabling recursive Bayesian updating remains insufficiently explored.

#### Research gap

In summary, existing integrated evaluation approaches have provided valuable insights but still face several limitations:

(1) Multi-criteria fusion methods depend heavily on expert knowledge, introducing subjectivity.

(2) Predictive models are constrained by data availability and generalization.

(3) Most fusion frameworks treat inspection and monitoring separately, without fully exploiting their complementary temporal and structural information. Furthermore, prior studies typically focus on fusing single-dimensional signals (e.g., displacement or acceleration)^14^ or simplified monthly averages, failing to capture the temporal dynamics and uncertainty interactions between inspection and continuous monitoring data.

#### Research aim

This study aims to develop a hierarchical Bayesian data fusion framework that unifies discrete inspection records and continuous monitoring data into a single probabilistic model for bridge deterioration analysis. The proposed approach leverages Bayesian inference and hierarchical modeling to achieve adaptive posterior updating, enabling both global risk quantification and real-time degradation tracking. Through this framework, the study seeks to establish a more interpretable, data-driven foundation for predictive bridge maintenance and reliability management.

## Methodology

### Overview

Building upon these advancements, this study proposes a hierarchical Bayesian deterioration model that integrates discrete inspection data with dynamic monitoring data to form a unified probabilistic degradation framework. This framework allows continuous updating of deterioration estimates as new monitoring data become available, enhancing both accuracy and adaptivity in bridge health assessment.

### Hierarchical bayesian model architecture

#### Model framework and update mechanism

The proposed three-layer hierarchical Bayesian model represents bridge deterioration across different informational hierarchies. The *Dynamic State Layer* captures time-varying behavior from continuous crack monitoring; the *Deterioration Risk Layer* interprets inspection-derived reliability; and the *Fusion Layer* performs posterior integration through Bayes’ theorem.

The overall joint posterior distribution is expressed as:

1 $$\:P\left({\Theta\:}\vert{D}_{i},{D}_{m}\right)\propto\:P\left({D}_{m}\vert{U}_{t},{\Theta\:}\right){\hspace{0.17em}}P\left({D}_{i}\vert{\Theta\:}\right){\hspace{0.17em}}P\left({\Theta\:}\right)$$

where $\:{D}_{i}$and $\:{D}_{m}$represent inspection and monitoring datasets, respectively.

**Fig. 1:**
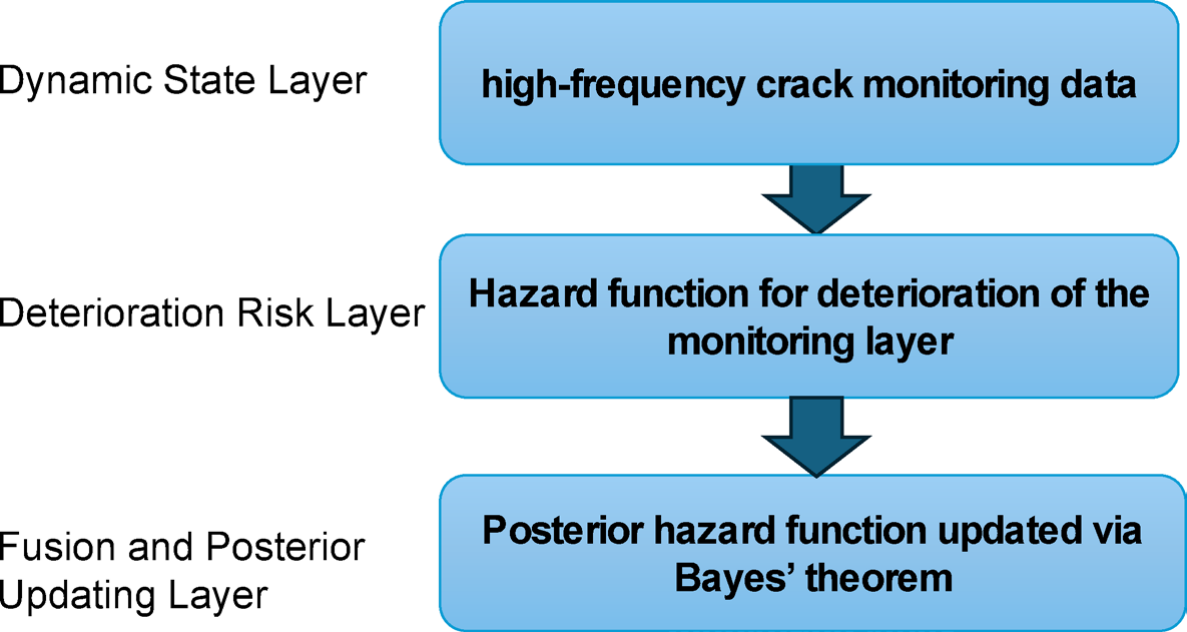
Framework of the proposed hierarchical Bayesian model.

The components of high-frequency crack monitoring data were first analyzed. A hazard function for deterioration of the monitoring layer was then established. Finally, this hazard function was continuously refined to obtain a posterior hazard function updated via Bayes’ theorem using failure probability by inspection information, the proposed framework of this study is illustrated in Fig. 1.

(1) Dynamic State Layer.

The first layer represents the dynamic state of the bridge, primarily derived from high-frequency crack monitoring data. Key observed variables include crack width evolution, temperature variation, and other time-dependent responses.

Let Ut denote a stochastic variable representing the physical state of the bridge at time t, expressed as:

2 $$\:{U}_{t}={u}_{1}\left(t\right),{u}_{2}\left(t\right)\dots\:{u}_{n}\left(t\right)$$

where each component $\:{u}_{i}\left(t\right)$represents a measurable or inferred deterioration-related response. Typical elements include crack width $\:w\left(t\right)$, temperature $\:T\left(t\right)$, crack opening rate $\:\dot{w}\left(t\right)$, or oher structural response indicators.

To explicitly model the stochastic evolution of crack width under environmental influence, the dynamic state variables in this layer are estimated using a Metropolis–Hastings (MH)-based Bayesian regression model.

Specifically, incremental crack growth is modeled as a function of time-varying temperature and historical crack width observations. Given the absence of closed-form posterior solutions due to nonlinear dependencies and measurement uncertainty, MH sampling is employed to infer the posterior distribution of crack growth parameters.

The resulting posterior samples are used to estimate the expected incremental crack growth, which is subsequently aggregated over monthly intervals to construct a continuous crack evolution trajectory. This trajectory constitutes the probabilistic realization of the dynamic state variable $\:{U}_{t}$and serves as the monitoring-based input to the upper fusion layer.

(2) Deterioration Risk Layer.

The second layer, termed the deterioration risk layer, transforms the discrete inspection ratings into a continuous deterioration risk function, which quantitatively represents the bridge’s degradation state inferred from inspection data.

According to John’s Bayesian formulation of structural reliability^36^, the prior distribution of the deterioration state variable U is defined as:

3 $$\:\pi\:\left(U\right)=\tau\:{e}^{-f\left(U,X\left(U\right)\right)}$$

where is a normalization constant ensuring that $\:\int\:\pi\:\left(U\right)dU\:$ and $\:f\left(U,X\left(U\right)\right)=\sum\:{\beta\:}_{i}g\left({\omega\:}_{i},{x}_{i}\left({u}_{i}\right)\right)\:$is an interactive deterioration potential function that characterizes the joint effect of crack attributes and environmental variables, which can be expressed as:

4 $$\:g\left({\omega\:}_{i},{x}_{i}\left({u}_{i}\right)\right)=\int\:{\left[{\beta\:}_{1}\right({L}_{i}\left(t\right)-{L}_{i}\left(t-1\right))}^{2}+{{\beta\:}_{2}\left(\varDelta\:{L}_{i}\left(t\right)-{\varDelta\:L}_{i}\left(t-1\right)\right)}^{2}+ϵ]dt$$

$\:{\omega\:}_{i}$represents the weighted deterioration index of the $\:i$-th crack (e.g., width, propagation rate, or severity),

$\:{\beta\:}_{i}$denotes the sensitivity coefficient associated with each deterioration factor, and.

$\:g\left({\omega\:}_{i},{x}_{i}\left({u}_{i}\right)\right)$is a localized deterioration response function, formulated as:

$\:{L}_{i}^{t}$and $\:{L}_{i}^{t-1}$denote the crack width at two consecutive inspection times, $\:{T}_{i}^{t}$is the corresponding temperature, and $\:\epsilon\:\:$is a stochastic noise term capturing unmodeled uncertainty.

(3) Fusion and Posterior Updating Layer.

The third layer serves as the information fusion and posterior updating layer, where the inspection-based risk model and the monitoring-based dynamic model are coupled within a Bayesian hierarchical structure., The posterior distribution $\:P\left(U\vert C\right)$is computed using Bayes’ Theorem, as shown in the following equation:

5 $$\:\begin{array}{cccc}&\:P\left(U\vert C\right)=\frac{P\left(C\vert U\right)\cdot\:\pi\:\left(U\right)}{\int\:P\left(C\vert U\right)\cdot\:\pi\:\left(U\right){\hspace{0.17em}}dU}&\:&\:\end{array}$$

Where: $\:P\left(C\vert U\right)$represents the likelihood, or the conditional probability of observing the deterioration condition $\:C\:$given the system state $\:U$from periodic inspection data. The denominator, $\:\int\:P\left(C\vert U\right)\cdot\:\pi\:\left(U\right){\hspace{0.17em}}dU$, is the normalizing constant, ensuring that the posterior distribution integrates to 1 over all possible values of $\:U$.In this framework, $\:P\left(C\vert U\right)$is computed using the Weibull-based Accelerated Failure Time (AFT) model, which models the deterioration process of the system.

6 $$\:P\left({U}_{t}\vert{C}_{1:t}\right)=\frac{P\left({C}_{t}\vert{U}_{t}\right)\hspace{0.17em}\:\left({U}_{t}\vert{C}_{1:t-1}\right)}{\int\:P\left({C}_{t}\vert{U}_{t}\right)\hspace{0.17em}P\left({U}_{t}\vert{C}_{1:t-1}\right)\hspace{0.17em}d{U}_{t}}$$

Here, $\:P({U}_{t}\mid\:{C}_{1:t-1})$denotes the posterior distribution from the previous updating step, which serves as the prior for the current update. This recursive formulation enables sequential Bayesian updating, allowing the deterioration state to be progressively refined as new observations become available.Weibull based Accelerated Failure Time (AFT) Model.

The AFT model is a survival analysis method that models the time until a failure event (e.g., a crack reaching a critical threshold). It is commonly used in reliability analysis to predict the time to failure based on covariates that influence deterioration processes. The AFT model assumes that the log-transformed failure times are linearly related to the covariates.

In our approach, the AFT model is used to quantify the relationship between bridge deterioration parameters (such as crack count, crack type, and environmental factors) and the failure time of the bridge components. Specifically, the AFT model is represented as:

7 $$\:\mathrm{log}\left(T\right)={\beta\:}_{0}+{\beta\:}_{1}{X}_{1}+{\beta\:}_{2}{X}_{2}+\cdots\:+{\beta\:}_{k}{X}_{k}+ϵ$$

Where: $\:T$is the time to failure (e.g., time to significant crack propagation), $\:{X}_{1},{X}_{2},\dots\:,{X}_{k}$are the covariates (such as crack type, temperature), $\:{\beta\:}_{0},{\beta\:}_{1},\dots\:,{\beta\:}_{k}$are the coefficients, $\:ϵ\:$represents the error term.

In the original Weibull deterioration density function, the hazard probability was defined as, according to Washer^37,38^:

8 $$\:f\left(t\right)=\alpha\:\gamma\:{t}^{\alpha\:-1}{e}^{-\gamma\:{\alpha\:}^{t}}$$

Following Collins’ formulation of Bayesian reliability analysis^33^, the prior distribution of the risk rate parameter is defined as:

9 $$\:Lambda\lambda\:\sim\:\mathrm{Gamma}\left({\alpha\:}_{0},{\beta\:}_{0}\right)$$

Therefore, $\:\mathrm{log}\lambda\:\left(U,\theta\:\right)=\mu\:+{X}^{{\top\:}}\beta\:+\gamma\:U$, $\:\mathrm{X}=\left({X}_{1},{X}_{2},...,{X}_{k}\right)$ ,$\:\beta\:=\left({\beta\:}_{0},{\beta\:}_{1},\dots\:,{\beta\:}_{k}\right)$ the likelihood of observed inspection data given deterioration time $\:T$is modeled using a Weibull AFT formulation:

10 $$\:P\left(C\vert U\right)=\alpha\:{t}^{\alpha\:-1}{e}^{-(\mu\:+{X}^{{\top\:}}\beta\:{)}}$$

Where the α is the shape parameter, following the log-normal distribution.β is a vector of regression coefficients for X, which represents the observation values (like crack type, damage component). µ is a baseline for the scale parameter. This component quantifies global deterioration risk by linking inspection observations to underlying structural reliability^39^. Although the inspection data are reported annually, forming a discrete-time sequence, they can be reasonably approximated as continuous variables through interpolation, a practice that has been formally validated in prior statistical research. In particular, Gordon Law and Brookmeyer (1987)^40^demonstrated that midpoint and related interpolation approaches yield unbiased or minimally biased estimates in doubly censored and interval-based datasets, thereby supporting the appropriateness of applying interpolation to discretely observed deterioration variables in this study. This transformation enables a more tractable computation process and facilitates the interpretation of long-term degradation trends. Consequently, both the failure probability and time were treated as continuous variables in the deterioration modeling process.

And the corresponding survival function:

11 $$\:S\left(t\right)=1-{\int\:}_{0}^{\mathrm{t}}P\left(C\vert U\right){\hspace{0.17em}}dt$$

To ensure continuity, $\:P\left(U\vert C\right)=1\:$is assumed for time periods prior to the onset of monitoring data. As new monitoring information becomes available, the posterior is updated adaptively, providing a dynamic, data-driven deterioration trajectory.

The likelihood function derived from the Weibull-based AFT model serves as the probabilistic foundation for Bayesian posterior inference, which is numerically implemented using the Metropolis–Hastings algorithm described in Sect. Metropolis–Hastings algorithm for posterior inference

### Posterior updating frequency

The posterior updating process is performed in a time-adaptive manner, driven by the availability of new monitoring or inspection data. Specifically, posterior updates are triggered whenever new valid observations are acquired, rather than at fixed time intervals. For continuous monitoring data, posterior updates are executed at the native sampling resolution of the monitoring system, while inspection-based updates are performed upon the arrival of each periodic inspection record.

This event-driven updating strategy allows the model to maintain high temporal resolution when monitoring data are available, while preserving consistency with the coarser inspection intervals. As a result, the deterioration state can be continuously refined as new information becomes available.

#### Influence of data measurement errors on the model

Sensor errors—such as noise, signal drift, or limited resolution—and inaccuracies in manual crack measurements can potentially introduce bias or variance into both the dynamic monitoring series and the periodic inspection records. In the proposed hierarchical Bayesian fusion framework, these uncertainties are explicitly modeled through layer-specific stochastic error terms. Prior distributions are assigned to the measurement-error variances of each data source, allowing the inference process to differentiate true deterioration signals from random noise. During posterior updating, high-uncertainty observations naturally receive reduced influence because the Bayesian weights are inversely related to their estimated variance. Furthermore, the integration of multi-resolution data (continuous monitoring and periodic inspections) provides cross-validation across sources, which helps to suppress spurious fluctuations arising from local measurement errors. Together, these mechanisms enable the model to mitigate sensor-related inaccuracies and preserve the robustness of the inferred deterioration trajectories.

Prior to posterior updating, all incoming data are subjected to a quality-screening procedure to determine their suitability for inference. This procedure includes checks for missing values, abnormal variability, and physically implausible measurements. Only observations satisfying predefined quality criteria are incorporated into the updating step.

In particular, observations exhibiting excessive variance, abrupt discontinuities inconsistent with known deterioration mechanisms, or prolonged data loss are either excluded from updating or assigned reduced statistical influence through inflated measurement-error variance. This quality-gated updating strategy ensures that posterior inference is driven primarily by reliable information, thereby preventing spurious updates caused by sensor malfunction or measurement noise.

### Computational implementation

#### Metropolis–Hastings algorithm for posterior inference

Parameter estimation within the proposed hierarchical Bayesian framework is performed using Markov Chain Monte Carlo (MCMC) sampling, implemented via the Metropolis–Hastings (MH) algorithm. It is emphasized that the MH algorithm does not constitute a deterioration model itself; rather, it serves as a numerical inference engine for sampling from the posterior distributions induced by the AFT-based likelihood and the specified prior distributions^41,42^.

The MH algorithm is employed to efficiently explore high-dimensional posterior spaces when closed-form solutions are not available. At each iteration, candidate values of the deterioration parameters $\:\left(\alpha\:,\beta\:,\mu\:\right)$are proposed and accepted or rejected based on the corresponding posterior density ratios. These parameters characterize different aspects of the deterioration process, including the temporal evolution rate of crack propagation and the baseline deterioration level of the bridge system.

Through iterative sampling, the MH procedure yields empirical posterior distributions of model parameters, which are subsequently used for posterior prediction and dynamic updating as new inspection or monitoring data become available.

#### Computational workflow

The computational workflow can be summarized as follows:

1. Input Data: Import two heterogeneous data sources:

(i) discrete inspection-based deterioration indicators used to characterize long-term structural risk, and.
(ii) continuous time-series monitoring data (e.g., crack width and temperature) used to capture short-term degradation dynamics.

2. 2. Prior Definition: Specify hyperparameters $\:\left({\alpha\:}_{0},{\beta\:}_{0},{\mu\:}_{0}\right)$of the Gamma prior distributions for the deterioration parameters governing both inspection-based risk evolution and monitoring-based crack growth dynamics.

Likelihood Construction: Construct source-specific likelihood functions:

(i) a Weibull AFT-based likelihood for discrete inspection data to model time-to-deterioration risk, and.
(ii) an exponential hazard–based likelihood (or equivalent regression likelihood) for monitoring-derived crack growth increments.

3. 3. Posterior Sampling: Perform Markov Chain Monte Carlo sampling using the Metropolis–Hastings algorithm to jointly sample posterior distributions of the deterioration parameters conditioned on both inspection and monitoring likelihoods.
4. 4. Posterior Prediction: As new monitoring observations become available, the previously obtained posterior distributions are propagated as priors for the subsequent updating cycle, allowing recursive refinement of deterioration parameters without reprocessing historical data.

**Fig. 2:**
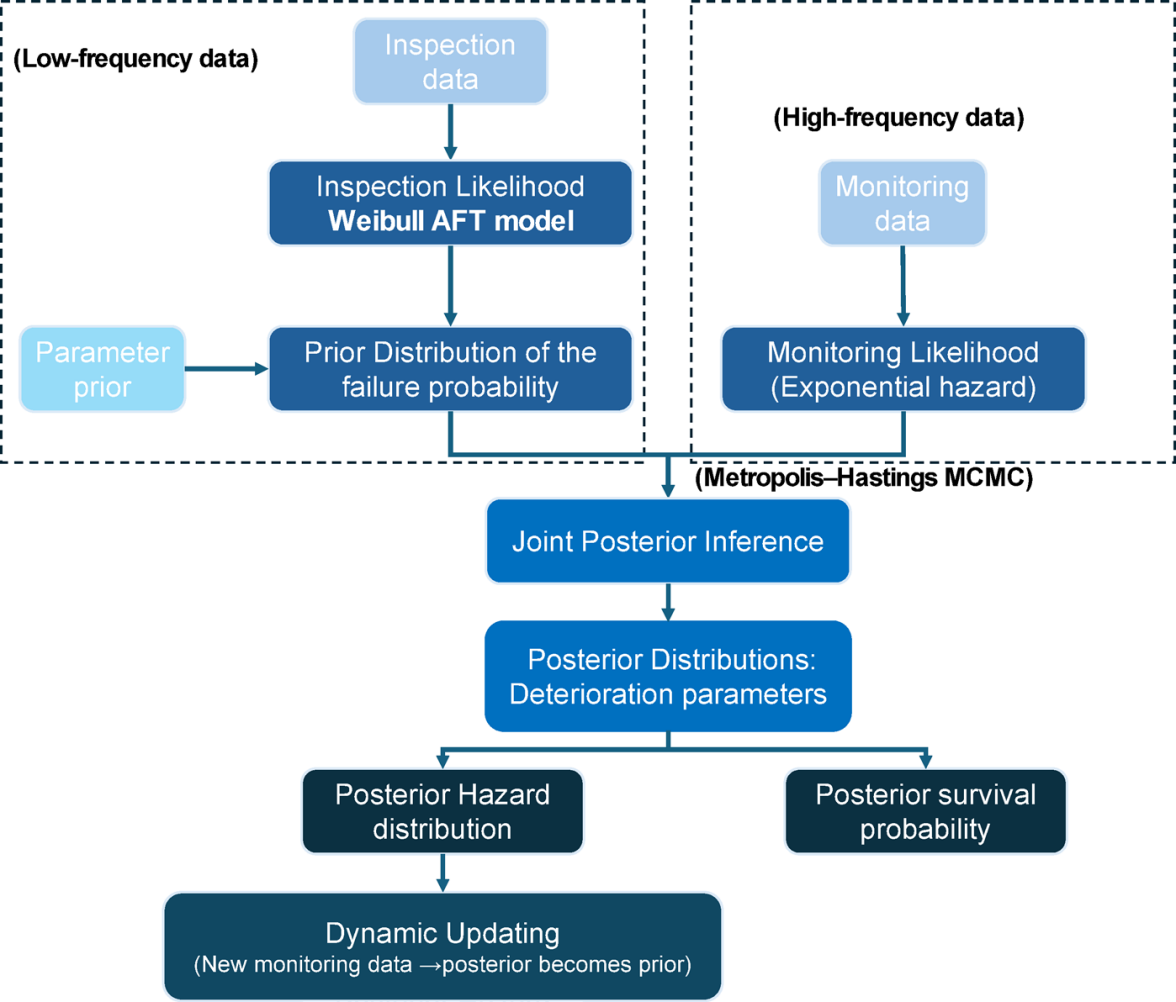
Bayesian updating framework integrating periodic inspection and continuous monitoring data.

The overall framework for integrating data and updating reliability assessments in fusion and posterior updating layer is illustrated in Fig. 2. This schematic outlines the Bayesian process of combining periodic inspection data with continuous monitoring streams to progressively update the posterior estimates of structural hazard and survival probability. This framework thus provides a self-adaptive deterioration inference system, capable of integrating asynchronous, multi-scale information for real-time health assessment.

At each updating step, the Metropolis–Hastings sampler is initialized using the posterior distribution obtained from the previous step, thereby ensuring continuity and computational efficiency in the sequential updating process.

## Case study and model validation

### Case study

#### Bridge description and structural characteristics

**Fig. 3:**
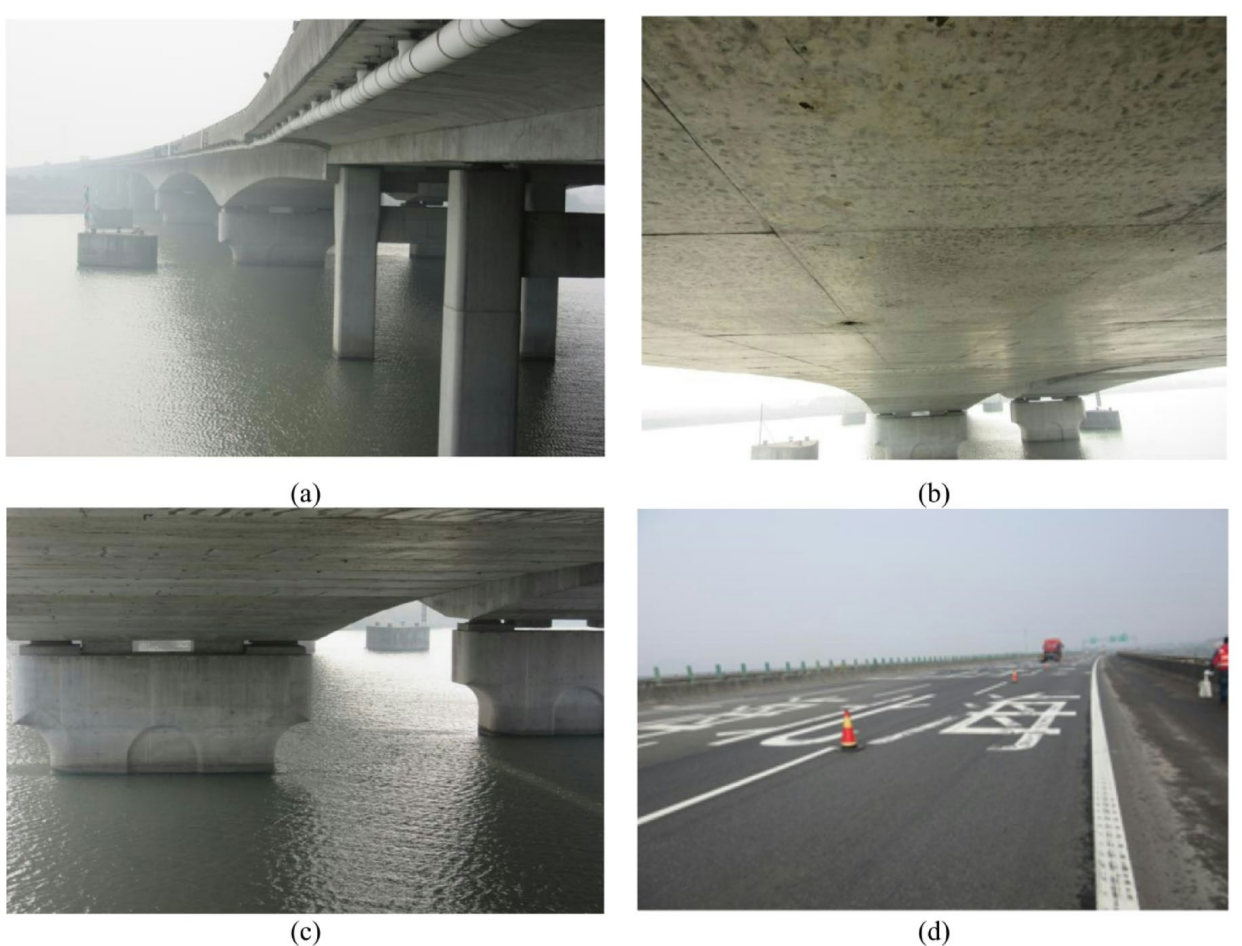
Photographs showing key components of the Fenghua River Bridge: (**a**) overall view; (**b**) superstructure; (**c**) substructure; (**d**) bridge deck.

The Fenghua River Bridge is a large-scale prestressed concrete continuous box girder bridge located at chainage K65 + 122.5, Ningbo City, Zhejiang Province, China, with a total length of 1,684.7 m. The main bridge, spanning 230 m, consists of three continuous variable-depth prestressed concrete box girders arranged in a (65 m + 100 m + 65 m) configuration, crossing the Fenghua River orthogonally. The external view of the bridge is depicted in Fig. 3.

The approach bridges are composed of 30 m cast-in-place box girders and a combination of 30 m and 20 m simply-supported hollow slab girders made continuous after construction. The detailed span arrangement is as follows:

##### Left carriageway

11 × 30 m + 20 m + 43 × 20 m + 3 × 30 m + 65 m + 100 m + 65 m + 3 × 30 m + 3 × 20 m.

##### Right carriageway

11 × 30 m + 8 m + 12 m + 43 × 20 m + 3 × 30 m + 65 m + 100 m + 65 m + 3 × 30 m + 3 × 20 m.

The substructure consists of column-type abutments supported on drilled cast-in-place concrete piles. Expansion joints are designed as both tooth-type (comb-type) and steel-type joints to accommodate longitudinal deformation.

**Fig. 4:**
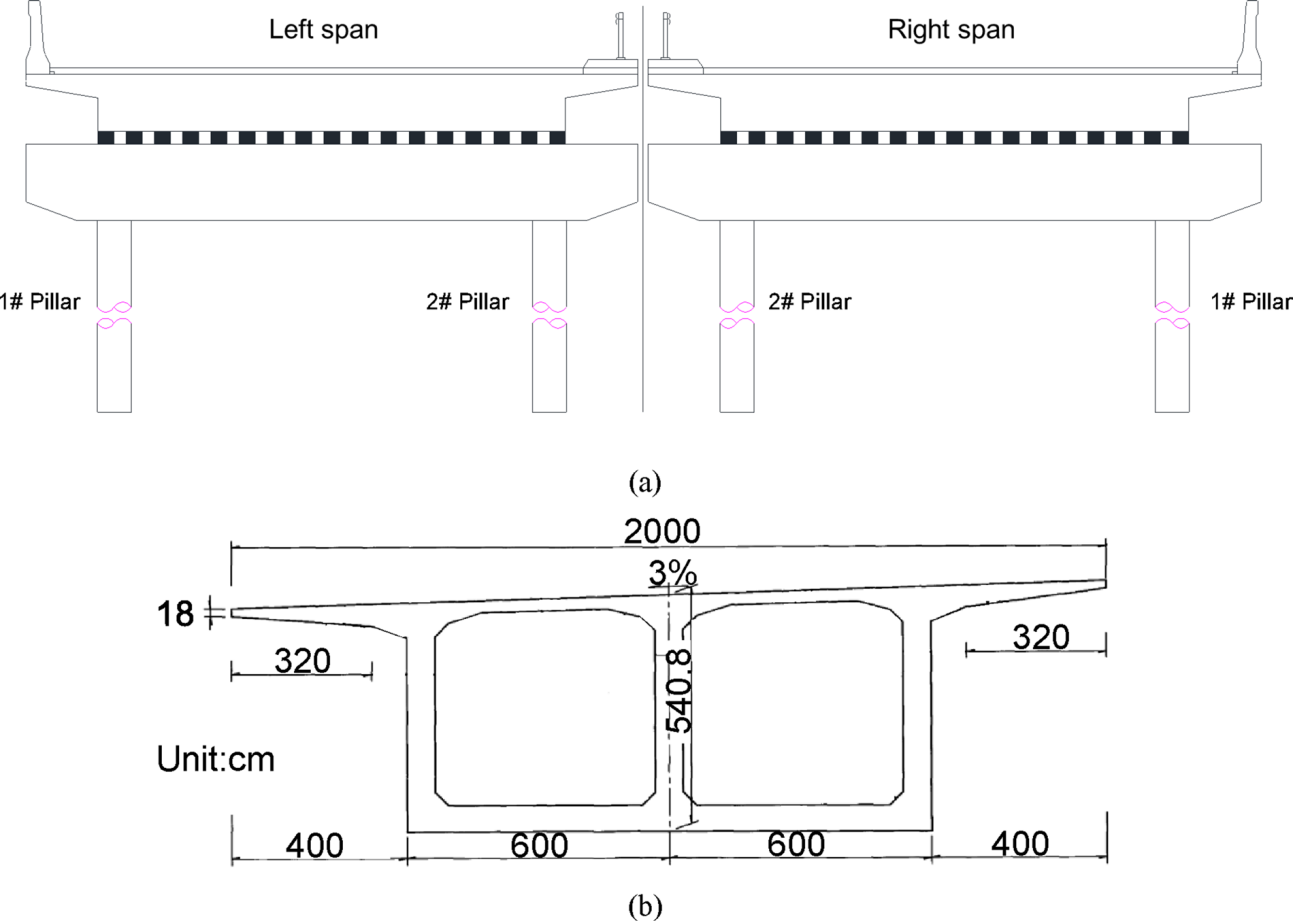
Cross-sectional configuration of the Fenghua River Bridge. (**a**) Cross-sectional schematic of the bridge, (**b**) Detailed cross-sectional design drawing.

The bridge is designed for Vehicle Load Class: Truck–Super-20, Trailer–120, in accordance with Chinese design specifications. The deck pavement adopts an asphalt concrete surfacing. The bridge carries a dual six-lane carriageway with a standard total width of 42 m, comprising two separated 19 m decks, providing bidirectional eight-lane traffic capacity, which is shown in Fig. 4.

#### Observed damage patterns and structural interpretation

**Fig. 5:**
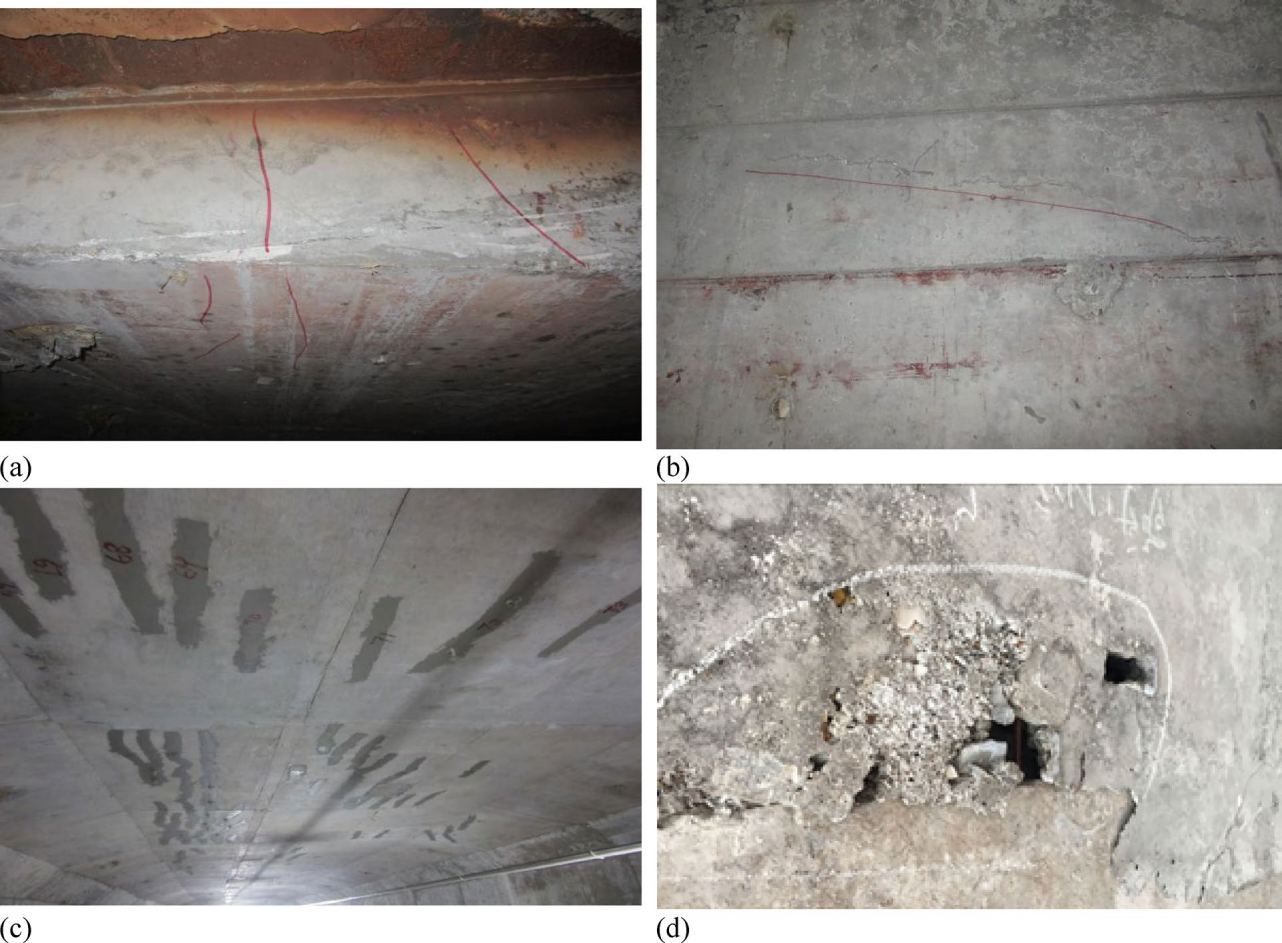
Typical structural distresses observed in Fenghua River Bridge. (**a**) Transverse crack on the roof slab, (**b**) Inclined crack on the web, (**c**) Longitudinal crack on the roof slab, (repaired), (**d**) Void and exposed reinforcement on the bottom slab.

The bridge has exhibited significant cracking within the box girder and beam components since 2010, resulting in a downgraded overall classification to Grade II. To more clearly illustrate the nature and extent of these deteriorations, representative field inspection photographs of the observed cracking, together with an overall view of the bridge, are provided in Fig. 5. These visual records were collected from the inspection data of year 2018 and offer a more intuitive understanding of the structural condition.

Figure 5 illustrates the typical cracking and damage patterns observed in the bridge, including transverse cracks in the roof slab, inclined cracks in the web, longitudinal cracks in the roof slab (partially repaired), and local concrete spalling with exposed reinforcement in the bottom slab. These damage forms are spatially distributed across the primary load-bearing components of the concrete box girder, indicating distinct deterioration mechanisms associated with different structural functions.

**Fig. 6:**
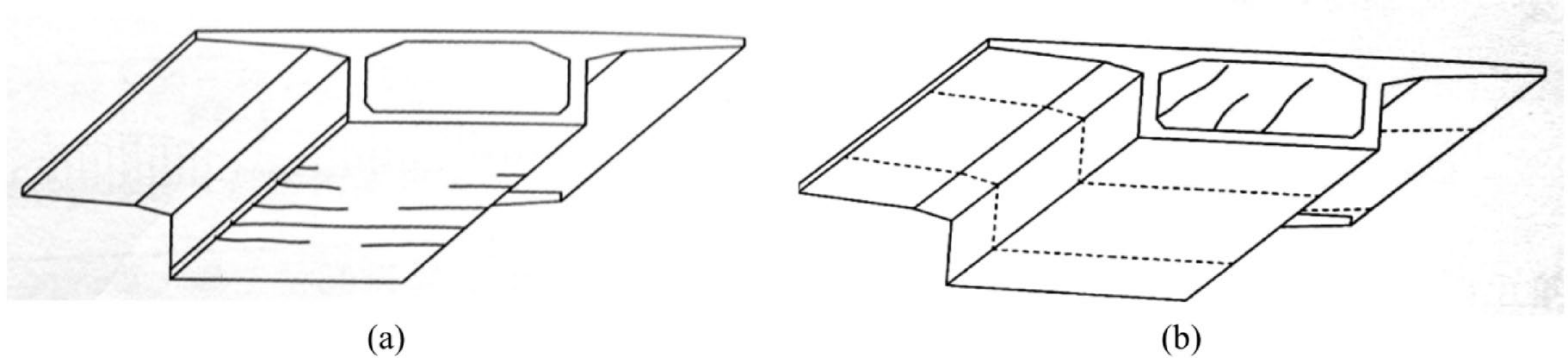
Schematic longitudinal cross-section of the concrete box girder illustrating the spatial relationship between observed cracking locations and structural components. (**a**) Transverse cracks in the bottom slab, (**b**) Longitudinal cracks in the roof slab.

Schematic (Fig. 6) showing the longitudinal cross-section of the concrete box girder and the damage observed within it.

Longitudinal cracks observed in the roof slab are primarily associated with the slab’s role as the upper flange of the box girder, which resists longitudinal bending induced by traffic loads and temperature gradients. Under global flexural action, the roof slab experiences tensile stresses along the bridge axis, particularly near regions of negative bending or restrained thermal deformation. Over time, repeated traffic loading and temperature-induced restraint can lead to the accumulation of tensile damage, resulting in longitudinal cracking aligned with the principal stress direction.

Transverse cracks and local concrete spalling in the bottom slab are indicative of tensile stresses perpendicular to the bridge axis, which arise from combined effects of global bending, shear transfer, and localized stress concentration. As the bottom slab functions as the lower flange of the box girder, it is particularly susceptible to tensile stress under sagging bending moments. Additionally, environmental exposure and moisture ingress can accelerate concrete degradation, leading to reinforcement corrosion and subsequent concrete cover loss.

The observed crack locations in Figs. 5 and 6 align well with the theoretical stress distribution of the box girder cross-section. Transverse cracking in the bottom slab and longitudinal cracking in the roof slab reflect the distinct tensile stress directions induced by global bending, while inclined web cracking corresponds to shear force transfer mechanisms. This spatial consistency confirms that the observed damage patterns are structurally driven rather than random or isolated defects.

#### Monitoring and inspection system configuration

**Fig. 7:**
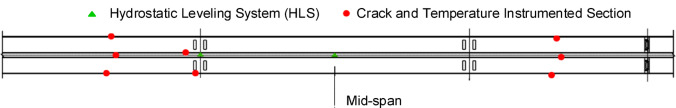
Schematic diagram of the sensor layout on the bridge (bird’s-eye view).

To enhance structural condition assessment, a remote crack monitoring system was installed in 2018, enabling long-term data collection and real-time deterioration tracking. To achieve the above monitoring objectives, the sensor system deployed on the bridge is illustrated in Fig. 7, primarily consisting of the following two types of devices: Crack and Temperature Instrumented Sections (CTIS) and Hydrostatic Leveling Systems (HLS). The former is used to track the long-term evolution of cracks while simultaneously acquiring structural temperature data, whereas the latter is mainly employed to monitor strain information in the bridge.

The detailed sensor configuration and sampling rates are summarized in Table 1.

**Table 1:** The detailed sensor configuration and sampling rates.

Num	Sensor ID	Sensor Type	Span	Position	Damage Type	Crack Type	Smapling Rate
1	12,081,814,187	CTIS	Right Span	13 m	Roof slab	Longitudinal crack	1 sample per hour
2	12,081,814,188	CTIS		40 m	Web	Longitudinal crack	
3	12,081,814,196	CTIS		51 m	Web	Inclined Crack	
4	12,081,814,192	CTIS		75 m	Web	Inclined Crack	
5	12,081,814,195	CTIS		84 m	Web	Inclined Crack	
6	12,081,814,203	CTIS		114.5 m	Roof slab	Longitudinal crack	
7	12,081,814,201	CTIS		115.5 m	Roof slab	Longitudinal crack	
8	12,081,814,202	CTIS		35 m	Web	Inclined Crack	
9	12,081,814,197	CTIS		51 m	Web	Inclined Crack	
10	12,081,814,198	CTIS		115 m	Roof slab	Longitudinal crack	
11	12,081,814,199	CTIS		121 m	Web	Inclined Crack	
12	12,221,902,218	HLS			Bridge End	Deflection Datum	
13	12,221,902,211	HLS			Mid-span	Deflection	
14	12,081,814,193	HLS			Bridge End	Deflection	
15	12,081,814,180	CTIS	Left Span	59 m	Web	Inclined Crack	
16	12,081,814,186	CTIS		32 m	Web	Inclined Crack	
17	12,081,814,181	CTIS		40 m	Web	Inclined Crack	
18	12,081,814,185	CTIS		72 m	Web	Inclined Crack	
19	12,081,814,183	CTIS		115 m	Roof slab	Longitudinal crack	
20	12,081,814,184	CTIS		103 m	Web	Inclined Crack	
21	12,081,814,179	CTIS		35 m	Web	Vertical Crack	
22	12,081,814,191	CTIS		77 m	Web	Inclined Crack	
23	12,081,814,190	CTIS		112 m	Web	Vertical Crack	
24	12,081,814,189	CTIS		115 m	Roof slab	Longitudinal crack	
25	12,081,814,178	CTIS		15 m	Roof slab	Longitudinal crack	
26	12,081,814,194	CTIS		56 m	Web	Inclined Crack	
27	12,221,902,217	HLS			Bridge End	Deflection Datum	
28	12,221,902,212	HLS			Mid-span	Deflection	
29	12,221,902,215	HLS			Bridge End	Deflection	

### Deterioration prediction and survival analysis

#### Data preprocessing

To validate the proposed integrated Bayesian model for crack evolution, two datasets were utilized:

(1) periodic inspection data of the Fenghua River Bridge collected from 2014 to 2023, and.

(2) continuous monitoring data acquired from 2023 to 2025.

**Fig. 8:**
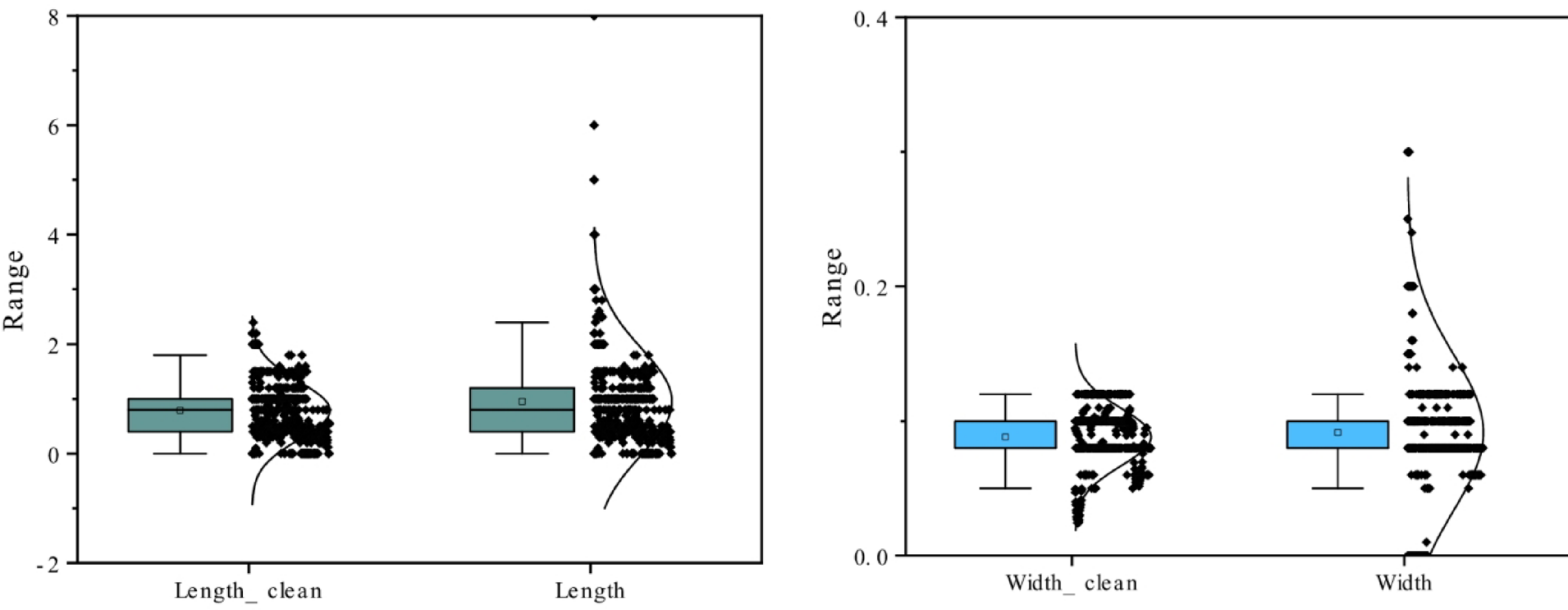
Boxplot of the comparison of the statistic distribution for the data before and after preprocessing.

All inspection and monitoring datasets underwent a structured preprocessing procedure. First, potential outliers in crack-geometry parameters were detected using a hybrid interquartile range (IQR) and median absolute deviation (MAD) criterion. Identified anomalies were then corrected using robust locally weighted regression to preserve the continuity and physical plausibility of crack-evolution characteristics. The boxplots of the raw data, illustrating the initial distribution and outlier patterns, are shown in Fig. 8.

Addtionally, multicollinearity among covariates in the survival model was examined using variance inflation factors (VIF). The result is summarized in Table 2. The VIF results are as follows:

**Table 2:** Multicollinearity assessment using variance inflation factors (VIFs) for inspection data.

Variable	VIF
const	4.578860
Crack Count	1.024594
Length	1.069437
Width	1.089248

All explanatory variables (Crack Count, Length, and Width) exhibit VIF values close to 1, indicating that no meaningful multicollinearity exists among the predictors. In general:

• VIF < 5 → No significant multicollinearity.

• VIF 5–10 → Moderate multicollinearity.

• VIF > 10 → Severe multicollinearity that may distort regression coefficients.

The constant term (const) shows a VIF = 4.578 and not relevant to multicollinearity assessment, since the intercept is not interpreted substantively.

These preprocessing steps ensure that the subsequent Bayesian inference and deterioration modeling are based on stable and reliable inputs.

#### Prior verification

In the inspection dataset, each recorded crack repair event was regarded as a failure occurrence, representing a discrete degradation episode in the bridge’s service life.

Prior to model construction, all input variables were encoded and discretized to ensure consistency across different data sources. The variables, their encoded values, and corresponding physical meanings are summarized in Table 3.

**Table 3:** Description and coding of variables used for crack characterization and structural inspection data.

Variable	Code	Description
Direction	1	Left (L)
	0	Right (R)
Damage Type	1	Roof slab
	2	Bottom slab
	3	Web
	4	Chamfer zone
	5	Tooth plate
	6	Closure segment
Crack Type	1	Longitudinal crack
	2	Transverse crack
	3	Inclined Crack
	4	Vertical Crack
Event Type	0	Observation
	1	Repair
Crack Count	Integer	Count of cracks identified in the inspected area
Length	Integer	Measured crack length (mm)
Width	Integer	Measured crack width (mm)

Subsequently, a Bayesian prior diagnostic was performed to evaluate the rationality of the assigned prior distributions for each model parameter. This step ensures that the priors are consistent with empirical observations and do not impose excessive bias on the posterior inference. The diagnostic results are presented as follows.

**Table 4:** Parameter estimates of the prior bayesian hierarchical deterioration model.

param	covariate	coef	exp(coef)	se(coef)	coef lower 95%	coef upper 95%	exp(coef) lower 95%	exp(coef) upper 95%
λ	crack_count	-0.07	0.94	0.09	-0.24	0.11	0.78	1.12
	crack_type_2	-0.52	0.59	0.28	-1.08	0.03	0.34	1.03
	crack_type_3	0.19	1.21	0.11	-0.03	0.41	0.97	1.5
	crack_type_4	0.07	1.08	0.12	-0.15	0.3	0.86	1.35
	damage_type_2	-0.27	0.76	0.2	-0.67	0.12	0.51	1.13
	damage_type_3	-0.08	0.93	0.09	-0.24	0.09	0.78	1.1
	damage_type_5	-0.11	0.9	0.14	-0.39	0.17	0.68	1.19
	damage_type_6	-0.74	0.48	0.27	-1.28	-0.21	0.28	0.81
	direction*	-0.54	0.58	0.09	-0.72	-0.37	0.49	0.69
	length	7.17	1301.03	7673998.02	-15040752.57	15040766.91	0	inf
	width	132.39	3.14E + 57	306959887.5	-601630191.9	601630456.7	0	inf
	Intercept	2.23	9.32	0.12	2	2.47	7.35	11.81
ρ	Intercept	1.35	3.85	0.1	1.15	1.55	3.16	4.7

*Note:“direction” indicates the crack location relative to the bridge span; left-span cracks are coded as the covariate level, and right-span cracks are used as the reference category.

The regression results in Table 4 indicate that the spatial location of cracks is a significant determinant of deterioration rate. In this model, the right-span cracks serve as the reference category, and the covariate span_direction is coded such that span_direction = 1 corresponds to left-span cracks (L). The estimated hazard ratio for left-span cracks is HR = 1.73 (95% CI: 1.46–2.05), indicating that cracks located in the left span exhibit a 73% higher deterioration risk compared with cracks in the right span. This significant spatial effect suggests a pronounced longitudinal dependence in the deterioration mechanism, likely associated with asymmetric loading patterns and environmental exposure conditions along the bridge alignment. In contrast, crack number, crack type, and most damage categories did not exhibit statistical significance within the model. Although the geometric parameters (length and width) showed extremely high estimated hazard ratios, their corresponding standard errors were substantial, leading to unstable confidence intervals. This instability implies potential multicollinearity or the presence of outliers, which warrants further investigation.

For crack_type_2, the estimated coefficient was − 0.52 with a p-value of 0.07—slightly above the conventional 0.05 significance threshold. Nevertheless, its exponential coefficient (exp(β) = 0.59) indicates a meaningful negative association with structural lifespan, suggesting borderline significance in practical terms.

#### Posterior inference and parameter Estimation

To further analyze the influence of these factors, the proposed risk-oriented deterioration model was applied to the periodic inspection data within a Bayesian posterior inference framework following Eqs. (8)-(9)^35,43^. The initial priorities for the key parameters were defined as follows:

$$\:\alpha\:\sim\:\mathrm{Lognormal}\left(\mathrm{0,1}\right),\beta\:\sim\:N\left(\mathrm{0,10}\right),\mu\:\sim\:N\left(\mathrm{0,10}\right)$$

Model parameters were estimated via Monte Carlo sampling, specifically using the MCMC algorithm for parameter calibration. The sampling configuration included 5,000 draws, 800 tuning steps, 4 parallel chains, and a target acceptance rate of 0.99, ensuring both convergence stability and sampling efficiency.

All computations were performed in Python 3.12 using the PyMC v5 library on a single-core CPU environment. To ensure reproducibility, the random seed was fixed at 42. The posterior summary statistics of the model parameters are provided in Table 5, which presents the posterior means, standard deviations, and 95% credible intervals for all estimated parameters^44^.

**Table 5:** Posterior summary statistics of the model parameters.

	mean	sd	hdi_3%	hdi_97%	mcse_mean	mcse_sd	ess_bulk	ess_tail	r_hat
alpha	38.102	0.58	37.016	39.207	0.005	0.003	15,277	13,841	1
mu	29.545	0.945	27.733	31.285	0.006	0.004	22,877	13,311	1
beta_count[0]	29.448	0.931	27.771	31.262	0.006	0.004	23,406	13,957	1
beta_count[1]	7.838	0.843	6.25	9.393	0.006	0.004	22,612	14,265	1
beta_count[2]	1.485	0.995	-0.362	3.389	0.006	0.005	23,673	14,408	1
beta_direction[0]	13.165	0.729	11.82	14.557	0.006	0.004	17,288	14,590	1
beta_direction[1]	16.367	0.725	15.013	17.744	0.005	0.004	17,583	14,359	1
beta_damage[1]	11.254	0.615	10.136	12.445	0.005	0.003	15,522	14,789	1
beta_damage[2]	0.269	0.891	-1.41	1.931	0.006	0.006	23,589	14,400	1
beta_damage[3]	9.62	0.655	8.373	10.831	0.005	0.004	16,330	14,790	1
beta_damage[4]	8.424	0.69	7.101	9.689	0.005	0.004	18,023	15,639	1
beta_damage[5]	-0.022	1.014	-1.838	1.98	0.006	0.008	28,815	14,260	1
beta_crack[1]	8.958	0.656	7.741	10.195	0.005	0.004	17,326	14,702	1
beta_crack[2]	3.044	0.71	1.698	4.373	0.005	0.004	19,801	16,179	1
beta_crack[3]	8.684	0.62	7.496	9.808	0.005	0.003	17,142	15,747	1
beta_crack[4]	8.858	0.582	7.742	9.93	0.005	0.003	15,667	15,311	1

Based on the posterior parameters obtained from the Bayesian inference procedure in Table 5, a corresponding bridge deterioration model was constructed to predict the evolution of structural lifespan under varying operating and environmental conditions.

**Fig. 9:**
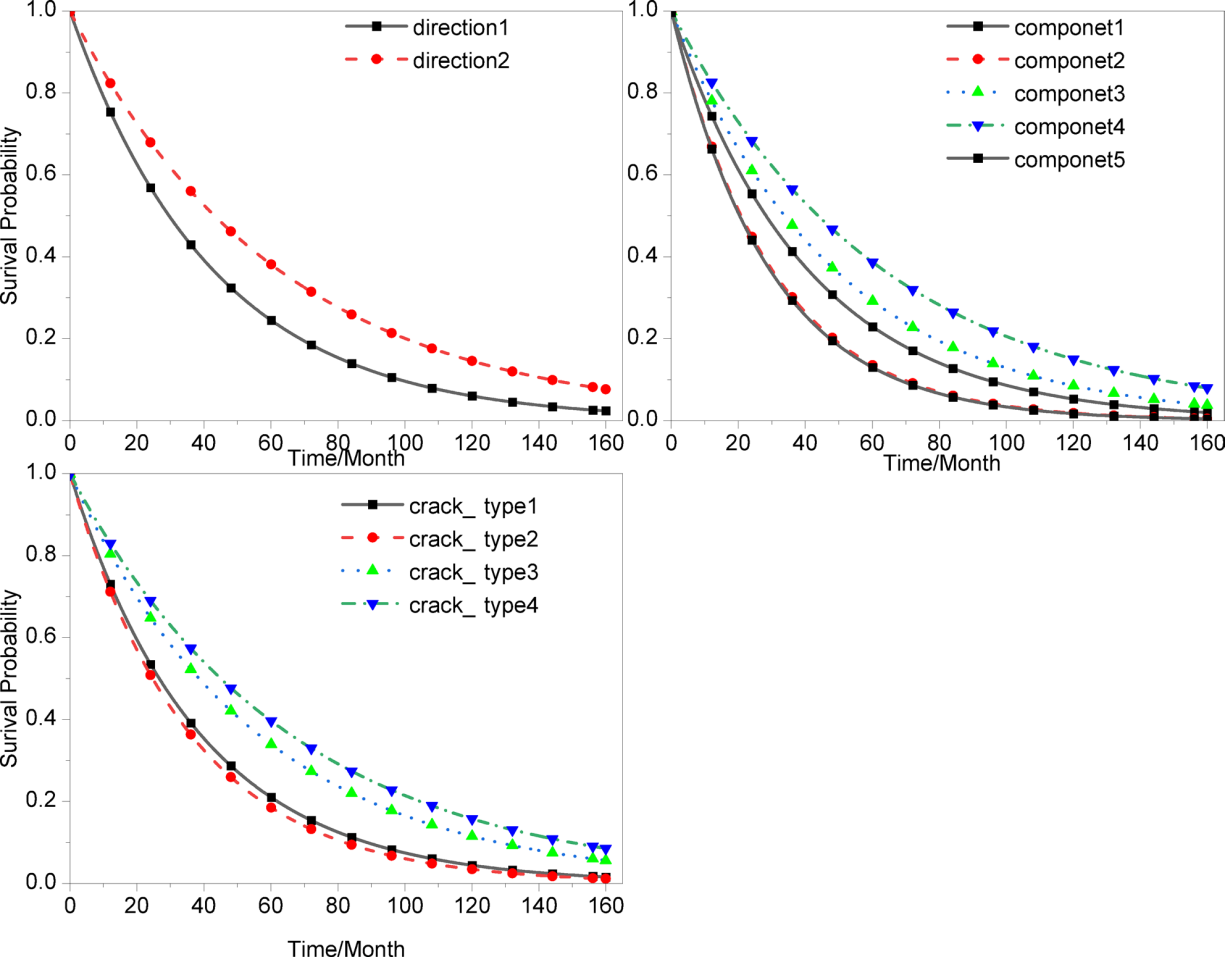
Comparison of survival probability for cracks with different conditions.

Figure 9 illustrates the predicted survival curves of the bridge under multiple structural and environmental states, where the horizontal axis represents time (months) and the vertical axis denotes the survival probability S(t). The figure captures the temporal evolution of the structural deterioration risk, offering clear insights into the degradation dynamics over time.

Several notable patterns can be observed:

Firstly, as shown in the upper-left panel of Fig. 9, survival curves corresponding to different crack orientations exhibit a consistent and pronounced separation throughout the observation period. Components classified as Orientation 2 demonstrate systematically higher survival probabilities compared to those with Orientation 1. This finding aligns with the Bayesian model estimates, which identified crack orientation as a statistically significant covariate. The result suggests that the structural or load asymmetry associated with Orientation 1 may accelerate the deterioration process, leading to a shorter expected service life.

Secondly, the component comparison panel (upper right in Fig. 9) reveals substantial heterogeneity in the deterioration behavior among different structural components. Certain elements—represented by the blue triangle-marked curve—maintain relatively high survival probabilities over time, whereas others exhibit a much steeper decline. This divergence implies that geometric configurations, local stress concentrations, or boundary constraints may induce varying vulnerability levels across components. Such heterogeneity underscores the necessity for component-specific inspection and mitigation strategies rather than a uniform maintenance approach, highlighting the potential benefit of risk-informed maintenance scheduling.

Lastly, the crack-type panel (bottom of Fig. 9) further demonstrates that distinct crack morphologies lead to markedly different degradation trajectories. In particular, crack type 2 exhibits the most adverse prognosis, characterized by the fastest decline in survival probability, whereas crack types 3 and 4 show relatively moderate deterioration rates. This observation corroborates the regression findings, where crack type 2 displayed a borderline-significant negative effect (exp(β) ≈ 0.59, *p* ≈ 0.07), indicating an approximately 41% reduction in expected lifespan compared to the reference category. These results emphasize the importance of prioritizing the inspection, reinforcement, and continuous monitoring of members exhibiting type 2 crack morphology.

Overall, the survival curves exhibit a two-phase deterioration pattern: a rapid decline in survival probability during the early service period (0–60 months), followed by a gradual stabilization phase. The median lifetime of members with left-direction cracks (Direction 1) is approximately 78 months, substantially lower than that of right-direction cracks (Direction 2, ≈ 110 months), confirming directionality as a dominant risk factor. Among all crack types, crack type 2 shows the lowest 60-month survival probability (S(60) = 0.43, 95% CI: 0.37–0.49), while crack type 4 performs comparatively better (S(60) = 0.58, 95% CI: 0.52–0.63). These quantitative results provide a probabilistic foundation for differentiated inspection intervals and targeted intervention policies.

### Monitoring-Based model updating

To refine the deterioration risk layer, the Metropolis–Hastings sampling algorithm was employed, consisting of two major stages^45^:

(1) Pilot Sampling.

A preliminary short-chain run of 5,000 iterations with independent normal proposals was first conducted to estimate the empirical covariance matrix of the parameter space. This covariance matrix was subsequently used to construct the multivariate proposal distribution for the main sampling phase.

(2) Main Sampling.

In the main phase, three independent Markov chains were executed, each running for 30,000 iterations using a multivariate normal random-walk proposal. To eliminate the impact of dimensional inconsistency among parameters, the input variable diff was standardized to have zero mean and unit variance. The cumulative integral of diff was then computed using the trapezoidal integration method, while linear interpolation ensured temporal continuity of the integral term at any time t.

To enforce the positivity constraint and enhance numerical stability, the scale parameter τ was log-transformed (log τ).

Convergence diagnostics were evaluated using the Gelman–Rubin statistic (Rhat) and the Effective Sample Size (ESS). All parameters achieved Rhat < 1.01, indicating satisfactory inter-chain mixing within the posterior space. The ESS values exceeded 1,000 for all parameters, demonstrating low autocorrelation and robust posterior estimates. The mean acceptance rate was approximately 0.38, falling within the recommended range (0.2–0.5) for random-walk M–H algorithms, thus ensuring an appropriate balance between exploration and acceptance.

**Fig. 10:**
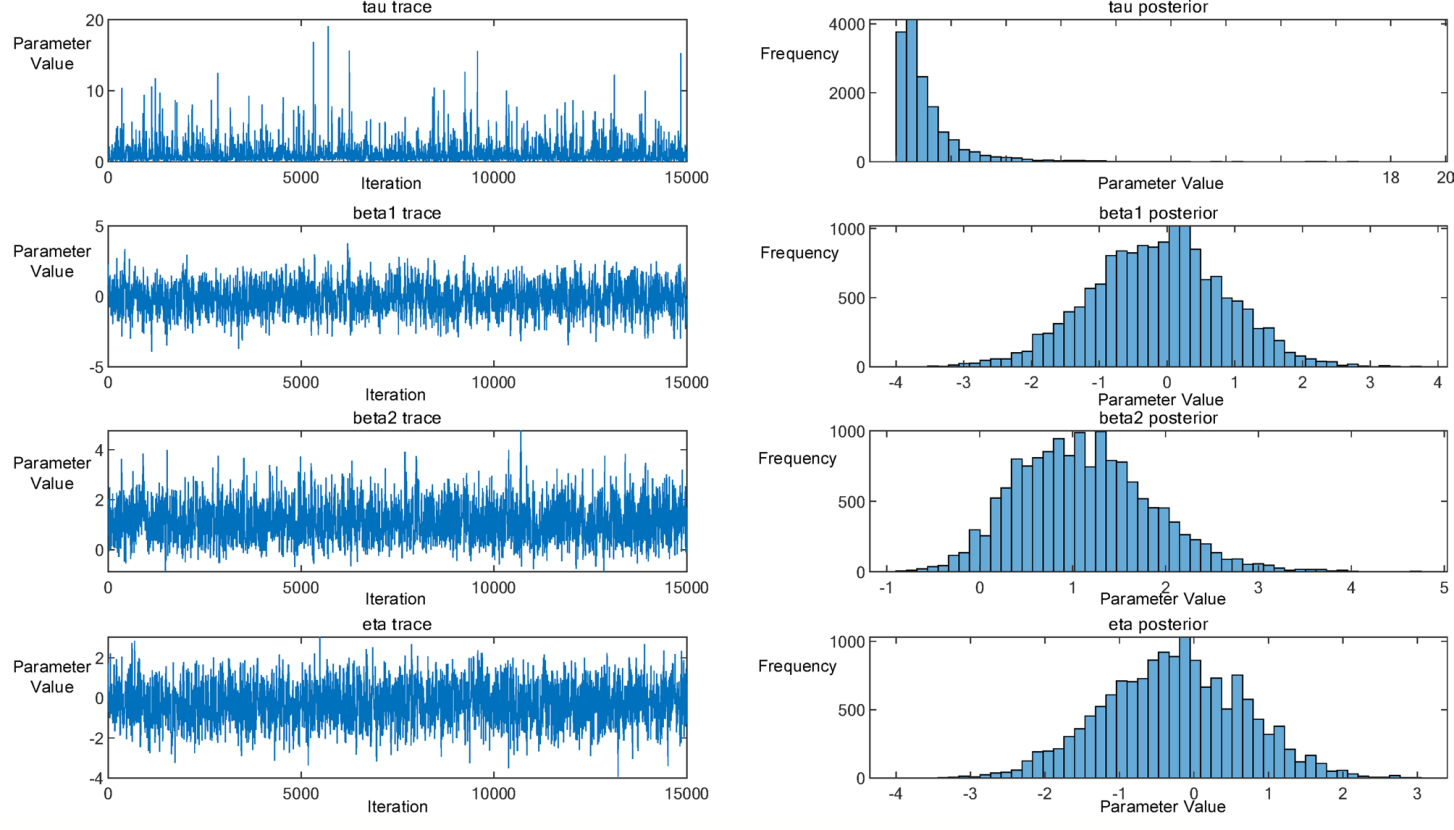
Posterior Bayesian trace of covariate and distributions for the estimated parameters.

As illustrated in Fig. 10, both the trace plots and posterior histograms exhibit stable distributions with unimodal characteristics, confirming the convergence and stability of the MCMC sampling process.

After convergence of the Markov chains, the posterior distribution analysis yielded the following median estimates for the key parameters:

τ = 0.71895, β₁ = −0.10858, β₂ = 1.0741, and η = −0.26553.

**Fig. 11:**
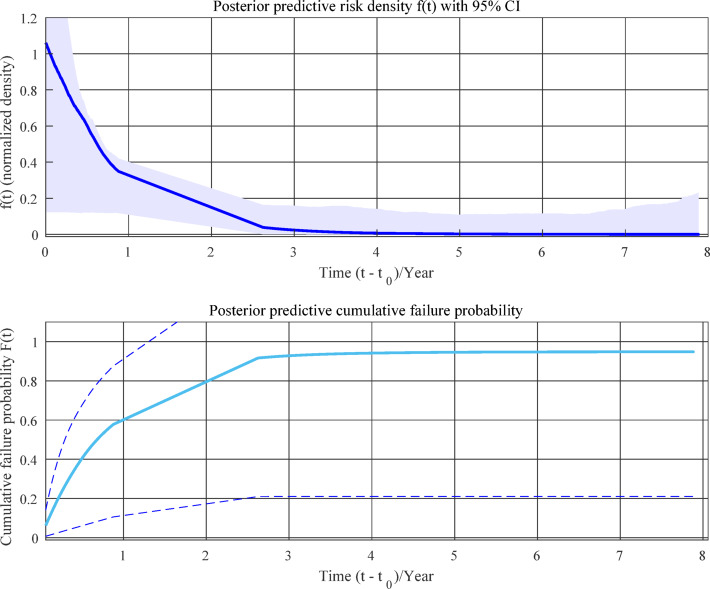
Posterior deterioration distribution and the cumulative hazard probability function of monitoring data.

Based on these estimates, the corresponding posterior deterioration distribution and the cumulative hazard probability function were subsequently derived in Fig. 11.

### Bayesian posterior updating and validation

#### Bayesian posterior updating and model performance

To integrate the monitoring information with the deterioration model established from periodic inspection data, a Bayesian updating framework is introduced in this study^44^.

The monitoring-based deterioration rate function $\:{f}_{\mathrm{mon}}\left(t\right)$serves as the likelihood term, reflecting the time-dependent degradation behavior observed during the monitoring period. According to Eq. (6), through Bayesian updating, the posterior deterioration probability function $\:{f}_{\mathrm{post}}\left(t\right)$can be derived.

This Bayesian fusion model dynamically integrates long-term inspection data with real-time monitoring information, enabling the deterioration model to simultaneously capture historical degradation trends and reflect the most recent structural condition. The updated posterior survival curve $\:{S}_{\mathrm{post}}\left(t\right)$describes the time-varying reliability evolution of the bridge structure, providing a more accurate and forward-looking basis for long-term health assessment and risk prediction.

**Fig. 12:**
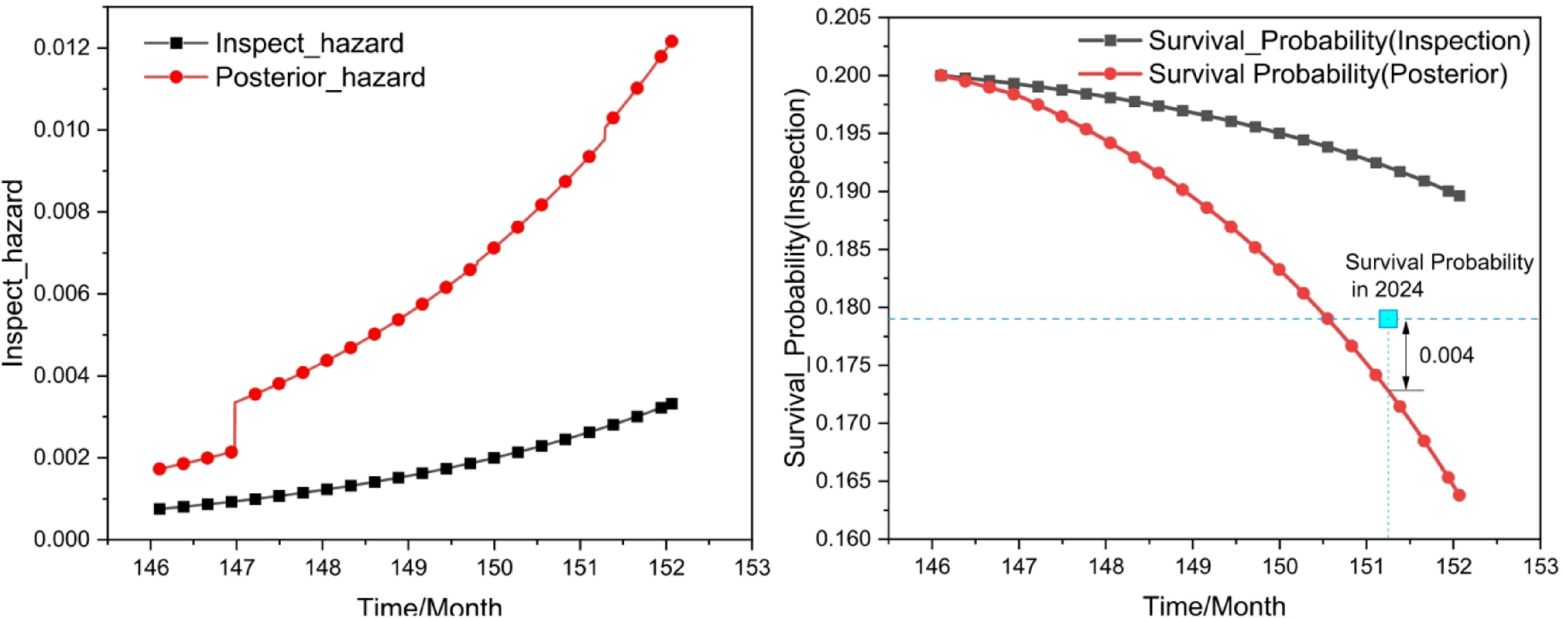
Posterior hazard function and survival probability curves obtained after Bayesian updating.

The following Fig. 12 illustrates the posterior hazard function and survival probability curves obtained after Bayesian updating, using the left span, Crack Type 1, and Damage Type 1 as representative examples.

As illustrated in Fig. 12, the deterioration rate significantly accelerates after Bayesian updating, exhibiting a posterior drift probability of approximately 0.004 within one year. This indicates that the updated model is more sensitive to the degradation signals reflected in the monitoring data, allowing early identification of potential risk evolution.

To rigorously assess the predictive performance of the proposed Bayesian framework, periodic inspection datasets from 2024 and 2025—neither of which were included in the model calibration stage—were employed for out-of-sample validation.

As summarized in Table 6, the posterior-updated predictions show close agreement with the observed deterioration probabilities for both validation years. Compared with the prior Weibull-AFT model, the posterior predictions consistently yield smaller absolute deviations from the observed inspection outcomes. Specifically, the absolute probability deviation of the posterior model remains below 0.01, whereas the prior model exhibits larger discrepancies, reaching up to 0.025.

These results indicate that posterior updating using monitoring-informed inference substantially improves predictive accuracy and enables more reliable tracking of year-to-year deterioration progression under previously unseen data.

**Table 6:** Out-of-sample validation of deterioration probability predictions using posterior updating and prior Weibull-AFT models.

Year	Observed Deterioration Probability	Predicted Probability		Absolute Deviation	
		posterior prediction	Weibull-AFT(Prior)	Posterior prediction	Weibull-AFT(Prior)
2024	0.176	0.172	0.194	0.004	0.018
2025	0.154	0.147	0.179	0.007	0.025

#### Diagnostic checks and model assumption verification

The model’s suitability was evaluated by comparing the predicted and observed distributions using p osterior predictive *p*- values

12 $$\:p=P\left(D\left({y}^{{\prime\:}},\theta\:\right)>D\left(y,\theta\:\right)\vert y\right)$$

where $\:{y}^{{\prime\:}}$is the model prediction, $\:y$the observed data, $\:theta\:the$ model parameters, and $\:D\left(\cdot\:\right)$the test statistic. A *p*-value near 0.5 indicates good agreement, while values close to 0 or 1 indicate significant deviation.

Residual independence was evaluated using the Durbin–Watson (D–W) test, which is suitable for time-indexed deterioration data because it detects potential temporal autocorrelation that may bias posterior updates. The obtained D–W posterior p-value was 0.67, indicating no statistically significant autocorrelation and confirming that the Bayesian updating mechanism did not introduce systematic temporal dependency.

Additionally, posterior predictive checks for normality, symmetry, and kurtosis all yielded p-values close to 0.5, suggesting minimal deviation from the assumed distributional properties and supporting the validity of the hierarchical model structure. 4.4.4 Quantitative Comparative Evaluation and Statistical Significance Testing.

To quantitatively assess predictive reliability, the proposed Bayesian fusion model was compared with two benchmarks:

(1) a Weibull-AFT model using only inspection data, which is the survival probability model without monitoring data updating and.

(2) a Cox-PH model with temperature as covariate. The Cox-PH model is a regression technique used to analyze survival data, where the hazard function is modeled as a function of covariates^29^. In this study, the Cox-PH model was applied to incorporate the effect of temperature as a covariate on the deterioration process. The model assumes that the hazard at any time is proportional to the baseline hazard, with the following form:

13 $$\:h\left(t\vert X\right)={h}_{0}\left(t\right)\cdot\:\mathrm{e}\mathrm{x}\mathrm{p}\left({\beta\:}_{1}{X}_{1}+{\beta\:}_{2}{X}_{2}+\cdots\:+{\beta\:}_{k}{X}_{k}\right)$$

Where: $\:h\left(t\vert X\right)$is the hazard function at time $\:t$, $\:{h}_{0}\left(t\right)$is the baseline hazard, $\:{X}_{1},{X}_{2},\dots\:,{X}_{k}$are covariates, and $\:{\beta\:}_{1},{\beta\:}_{2},\dots\:,{\beta\:}_{k}$are the coefficients.

Table 7 summarizes the performance metrics.

**Table 7:** Comparison of the performance of deterioration models.

Model	RMSE	D-W *p*-value	Coverage(95% CI)
Weibull-AFT	0.117	0.32	88.5%
Cox-PH	0.174	0.39	89.1%
Proposed Hierarchical Bayesian	0.0406	0.67	92.6%

The proposed model exhibits the lowest prediction error and the highest credible-interval coverage, indicating superior consistency between predicted and observed deterioration states. To evaluate whether performance differences were statistically significant, pairwise comparisons were conducted:

The reduction in RMSE achieved by the proposed model relative to both baseline models was statistically significant (*p* < 0.01) based on a paired bootstrap test.

The improvement in credible-interval coverage (92.6% vs. ~89%) was significant at the 95% confidence level based on a difference-in-proportions test.

The proposed model’s residual independence (*p* = 0.67) is significantly better than that of the baseline models (*p* ≈ 0.3).

These results confirm that the performance gains are not only numerically superior but also statistically meaningful, validating the model’s enhanced capacity to capture deterioration dynamics.

## Conclusion

Building upon conventional bridge deterioration models, this study develops a hierarchical Bayesian inference–based crack evolution fusion model, in which inspection-derived deterioration parameters and monitoring-based crack observations are organized into different inference levels, to integrate periodic inspection data with continuous monitoring measurements. By unifying information from different temporal resolutions and uncertainty levels, the proposed framework enhances the accuracy of deterioration characterization relative to inspection-only models, while enabling adaptive updating of deterioration states as new monitoring data become available. Application to the latest inspection and monitoring records demonstrates that the Bayesian-updated model provides closer agreement with observed crack propagation and time-dependent degradation trends than traditional inspection-only approaches. The model continuously updates deterioration parameters with newly available monitoring observations, reduces prediction uncertainty, and captures nonlinear evolutionary behavior, thereby improving the reliability of condition assessment and remaining service life prediction. Overall, this research provides a probabilistic, data-driven foundation for more informed and proactive bridge maintenance decision-making.

Despite these advantages, several limitations should be acknowledged. First, the current monitoring system is constrained by sensor coverage and measured variables at the observation level, with a primary focus on crack width. This limits the model’s ability to fully characterize underlying structural mechanisms or to distinguish between environmentally driven fluctuations and mechanically induced deterioration based solely on crack-width observations. Second, the framework assumes a relatively stable deterioration trajectory over time; its applicability may be reduced for bridge structures subjected to abrupt damage events or exhibiting rapid, localized deterioration that continuous monitoring does not fully capture. Third, the model relies on the availability of long-term, high-quality monitoring data; bridges without continuous sensing or with significant data gaps may require additional calibration or complementary modeling strategies.

Future research will address these limitations by integrating additional monitoring indicators—such as strain, displacement, temperature, and environmental effects—at the observation layer, and by expanding the framework into a multi-level, multi-source Bayesian network capable of modeling interactions among structural responses, environmental loads, and degradation processes. These extensions will enhance robustness, broaden applicability to diverse bridge types, and support a more holistic understanding of long-term structural performance.

## Data Availability

The code and dataset can be accessed at: [https://zenodo.org/records/17431883?token=eyJhbGciOiJIUzUxMiJ9.eyJpZCI6IjZmYmMwODNjLWM5YzUtNGQ3Ni04MTdhLTg1NjdhNGQzNjdlMyIsImRhdGEiOnt9LCJyYW5kb20iOiJkNjYyNDRmZTg3NzkxNzQ2YWRmMDZmMmZkMGIwZDA4NiJ9.GQ6h9FGqMlqUsRnSGQjGcmYbb7hvQOUID8GKCwvjtER7jvSD_9OxiT-vE5CIQurCojSwpz5tV5fXIdCiySNhfw]. Researchers requiring access to the raw dataset for validation under confidentiality agreements may contact the corresponding author to discuss a controlled-access mechanism.
